# Immunization against A/H1N1 pandemic flu (2009–2010) in pediatric patients at risk. What might be the most effective strategy? The experience of an health district of Northern Italy

**DOI:** 10.1186/1824-7288-38-18

**Published:** 2012-05-17

**Authors:** Giuseppe Gregori, Fabio Faccini, Ermanno Bongiorni, Ilario Maffini, Roberto Sacchetti

**Affiliations:** 1 Primary Care Pediatrician, Local Health Unit (AUSL), Piacenza, Italy; 2 Public Health Department, Local Health Unit (AUSL), Piacenza, Italy; 3 Primary Care Department, Local Health Unit (AUSL), Piacenza, Italy

**Keywords:** Pandemic influenza, Immunization strategy, Pediatric patients

## Abstract

**Background:**

Vaccination coverage rates against pandemic flu were far below those required by Italian Public Health Authorities.

The aim of this retrospective study was to assess how the management of vaccination against pandemic flu in the Health District of Piacenza (Northern Italy) had conditioned the adherence of patients at risk to the H1N1flu immunization program.

**Methods:**

From a population of 27.018 children aged between 6 months and 16 years, 2361 pediatric patients considered at risk according to the guidelines of the Ministry of Health were enrolled to receive pandemic flu vaccination.

Children enrolled in the immunization program were vaccinated with one of the following three options:

A) by their pediatrician in his office after contacting him directly or by phone

B) by their pediatrician in his office or in a public Health District office with the assistance of a nurse after an appointment had been booked by patient’s parents using a dedicated free of charge phone number

C) by a doctor of the public Health District after an appointment had been booked as for option B

**Results:**

The best outcomes of population vaccination coverage for pandemic flu were achieved when patients were vaccinated with option B (44.2%). For options A and C rates coverage results were 22.8% (OR 2,69) and 24.9% (OR 2, 39) respectively.

**Conclusion:**

The results of this study may be taken into account by the public health Authorities when planning the management of future immunization campaigns out of the usual vaccination schedule or in an emergency event.

## Background

In Italy the vaccination schedule is usually performed by the public Health District (HD) with exception of the seasonal flu vaccination which is carried out by the General Practitioner on adults considered at risk and over 65 years of age. There is not specific vaccination program in place for children at risk of pandemic flu. This vaccination is performed either by family, hospital or public HD doctors: only in a few HDs primary care pediatricians (PCPs) are involved systematically in the vaccination program.

Vaccination coverage rates against pandemic flu were far below those required by Italian Public Health Authorities: at the end of March 2010 less than 1 million people had been vaccinated in Italy [1].

In 2010, when pandemic flu started the main problems in order to obtain an effective population protection were:

1) To achieve a fast immunization of patients at risk, healthcare workers and specially staff working in intensive care units at hospitals.

2) To have the availability of adequate doses of vaccine before the beginning of the vaccination campaign.

To resolve the first problem, in the HD of Piacenza PCPs were involved in carrying out vaccination of pediatric patients at risk, the same as it happened in the past 10 years for seasonal flu vaccination programs. This choice was made because PCPs result more effective in convincing parents to vaccinate their children, because of their popularity among Italian families [2].

The second problem was unsolvable: the lack of adequate amount of vaccine before the peak of the pandemic flu reduced the number of subjects who underwent vaccination.

## Methods

### Background

Since 2000 PCPs working in the HD of Piacenza vaccinated against seasonal flu their patients after they were recognized to be at risk according to the guidelines of the Ministry of Health.

Vaccines were provided free of charge and patients were immunized between November and December every year.

Between 2000 and 2008 children at risk were enrolled in the seasonal flu vaccination program by their PCP. Parents were advised to vaccinate their children during routine visits or by consultation on the phone.

### Vaccination campaign against A/ H1N1 virus ( 2009–10)

In September 2009 each PCP (30 PCPs in total) identified pediatric subjects considered at risk following the guidelines of the Ministry of Health* [3].

In a population of 27.018 children aged between 6 months and 16 years, 2361 pediatric patients were enrolled to receive pandemic flu vaccination (PFV).

To perform vaccination PCP had to choose one of the following options:

A). To vaccinate patients in his office after previous appointment was taken by children’s parents on the phone or directly at the practice.

B). To vaccinate patients in his office or in a public HD office, with the assistance of a qualified nurse, after previous phone appointment using a dedicated free of charge phone number was booked by patients’ parents.

C). To delegate vaccination to a doctor of the public HD, after previous appointment using a dedicated free of charge phone number was taken by parents.

In our study 19 PCPs chose option A, 4 PCPs chose option B and 7 PCPs chose option C.

At the end of October 2009, near the beginning of vaccination campaign, the parents of children participating to the vaccination program were sent a letter signed by their PCP and agreed with the HD authorities: this letter remarked the usefulness of receiving PFV and which of the options available to perform the vaccination had been chosen by their PCP.

Anti A/H1N1 vaccine Focetria was used in pre-filled syringe 0.5 ml dose of thiomersal-free.

Written informed consent was obtained prior to vaccination. The vaccination campaign started on November 3, 2009 and ended on December 18, 2009. Statistical analysis was performed with chi-square test and O.R.

*chronic respiratory tract diseases, including asthma, bronchodysplasia, cystic fibrosis ;severe cardiovascular diseases, including congenital and acquired heart disease; diabetes mellitus and other metabolic diseases; severe liver disease and cirrhosis; kidney disease with renal failure; hematopoietic diseases and hemoglobinopathies;cancer; congenital and acquired diseases involving inadequate antibody production; immunosuppression induced by drugs therapy or HIV; chronic inflammatory intestinal diseases and malabsorption syndrome; diseases associated with an increased risk of aspiration such as neuromuscular diseases; obesity with body mass index (BMI) > 30; family member or close contact with persons at high risk for temporary or permanent contraindications for flu vaccination.

## Results

In this retrospective study 653/2361 (27.7%) children at risk underwent PFV. As shown in Table 1, best coverage rates for vaccination of patients considered at risk were achieved when PCPs chose to vaccinate patients with option B.

**Table 1:** Main data about pediatric population enrolled to receiving vaccination against A/H1N1 pandemic flu in the health district of Piacenza

	**Patients**	**At risk(%)**	**Vaccinated (%)**		**Primary Care Pediatricians**
Total	27018	2361 (8.7)	653	27.7	30
group A	16877	1510 (8.9)	344*	(22.8)	19
group B	3694	502 (13.6)	222	(44.2)	4
group C	6447	349 (5.4)	87 **	(24.9)	7

*group B vs group A : χ^2^ 85,63 p < 0.0000001 ; O.R. 2,69 (2,16-3,34).

** group B vs. group C : χ^2^ 33,1 p < 0.000001 ; O.R. 2,39 (1,75-3,26).

When PCP decided to vaccinate his patients choosing option B, children who underwent PFV vaccination were 44.2% of patients at risk.

Patients vaccinated using option A and C were 22.8% and 24.9% of total at risk respectively. The differences found were highly significant related to group B vs group A (χ2 85,63 p <0.000001; O.R. 2,69 [2,16-3,34] )and related to group B vs group C (χ2 33,1 p < 0.000001; O.R 2,39 [1,75-3,26]).

Data also shown that pediatric patients at risk enrolled for vaccination with option B were significantly more numerous (13.6%) when compared to others vaccinated with option A and C (respectively 8.9% and 5.4% ). Children vaccinated by PCP who chose for option B had a probability 1,6-2,75 fold higher to be enrolled for vaccination compared to other groups (Table 2)..

**Table 2:** Probability to be classified as patient at risk based on group membership

	**Children at risk**	**Children without risk**
Group B	502	3192
Group A	1510 *	15367
Group C	349 **	6098

* B vs A : χ2 74,02 p < 0,0000001 ; O.R 1,6 (1,44 – 1,78).

** B vs C : χ^2^ 204,2 p < 0,0000001 ; O.R. 2,75 (2,38 – 3,18)

## Discussion

According to the results of our study the involvement of PCP alone in performing PVF was not enough to maximize the adherence of patients to the vaccination program. The best coverage rate of vaccination for patients considered at risk for pandemic flu was achieved when PCP opted for option B, vaccinating patients in his office, with the assistance of a nurse, after children’s parents had booked an appointment calling a free of charge phone number (44.2%). Results achieved for patient vaccinated with option A (PCP performing vaccination alone) and C (public HD doctors performing vaccination) were similar, being 22.8% and 24.9% respectively. Authors suspect that the difference noted for vaccination coverage rates among the groups was mainly due ti two factors.

First we suggest that the availability of a dedicated free of charge phone number may have encouraged parents to book a vaccination appointment for their children because easy and quick. Second the availability of a nurse on premises likely allowed PCPs to save time and increase the number of patients immunized: nurses was actively involved in performing medical procedures and vaccinations making more efficient the activity of the pediatrician.

In our HD the vaccination coverage rate against pandemic flu (27.7%) was far lower from the years before regarding seasonal flu: in 2008 immunization coverage against seasonal flu in children at risk was 74.7% (1688/2258)(database of Piacenza HD, not published data).

PFV coverage rate was similar in France in the same target population [4]. We suggest that the early presentation of pandemic flu and the lack of an adequate amount of vaccine doses before the beginning of the vaccination campaign, had a negative effect on the adherence of patients to the vaccination program.

In Piacenza HD vaccination program started on the 9th of November 2009 because the PFV became available only from the end of October 2009 .The highest incidence of influenza A/H1N1 was reached between the 26th of October 2009 and the 23rd of November 2009. The peak consisted of > 50 new cases/1000 persons/ week in the range 0–14 years), with a peak at 46th week of the year [1].

We hypothesize that some emotional factors (e.g. newspapers headlines, inaccurate or confusing informations reported by mass media, different opinions and behaviors among healthcare workers, presence of adjuvant in PFV) played a negative effect on performances of vaccination program .It was not possible to assess properly this issue because no specific questionnaire in order to investigate the reasons of their choice was proposed to parents who decided to refuse the vaccination of their children.

Some Authors in countries other than Italy reported the low acceptability of A/H1N1 vaccination due to different reasons including fear of side-effects, vaccine safety, not believing in vaccinations or specifically in flu vaccination [5-8].

Our study suggests that coverage levels of vaccination against pandemic flu in children at risk in Piacenza HD could be considered satisfactory (27.7%) when compared to results obtained in other groups of patients in Italy. The vaccination coverage rate achieved in Italy for severely preterm infants younger than 2 years was 7.7% and for people at risk between the ages of 6 months to 65 years was 12.7% [1]. Another question arises about why PCPs who opted for option B enrolled a significantly higher number of patients at risk (13.6%, see Table 2). One possible explanation could be that the criteria used in selecting children at risk were not uniform among group A, B and C especially concerning asthma, the most frequent chronic disease of pediatric patients in Italy.

Being the prevalence of lifetime asthma 9.3% in italian children and 10.3% in adolescents [9] and considering that in Piacenza HD the prevalence of asthma is similar (9.5%) [10] we hypothesize that PCPs of group A enrolled less patiets for PFV (8.9%) because they chose strict selection criteria at the time of enrolling patients,especially with regards to asthma. This may have happened because, arranging appointments and performing vaccinations on their own, these PCPs were concerned about having an excessive work load and being unable to manage it successfully. It is possible that also PCPs who decided to not vaccinate personally their patients (option C) have used stricter selection criteria to include in the vaccination program patients with asthma and they were more reluctant to book their patients for PFV performed by a public HD doctor (only 5.4% of patients at risk).

We were unable to fully answer these questions because of the lack of data. Studies on this topics would be needed.

## Conclusions

Our retrospective study suggests that the presence of PCP, nursing support staff and the availability of a dedicated free of charge phone line for booking vaccination appointments seems to ensure the best outcomes for achieving a wider coverage immunization against pandemic flu in pediatric patients.

The pandemic flu is a public health issue priority and therefore public health experts play an essential role [11]. They have the responsibility to evaluate the impact of public health interventions in the short and long term, taking into account the human and economic resources available, the ethical aspects of the individual and the society.

The results of our study may be considered by Public Health Authorities at the time of planning the management of immunization campaigns, especially in an emergency event or when these are not part of the usual vaccination schedule.

## Competing interests

The authors declare that they have no competing interests.

## Authors’ contributions

GG designed the study and has made substantial contributions in drafting manuscript, FF contributed to interpretation of data and performed statistical analysis, IM performed data acquisition and validation for every pediatrician partecipating to the study ,EB and RS verified data analysis and revised the manuscript critically. All authors read and approved the final manuscript.

## References

[B1] Influnet http://www.epicentro.iss.it/focus/h1n1/archivioflunews.asp-Flunews

[B2] Greco L Un miracolo solo italiano: una sanità di qualità a costo da saldi Il Medico Pediatra 2002 6 443 448

[B3] Ministry of Health Misure urgenti in materia di profilassi vaccinale dell’influenza pandemica A(H1N1) Ordinanza 11 settembre 2009 http://www.normativasanitaria.it/jsp

[B4] Tuppin P Samson S Weill A Ricordeau P Allemand H Seasonal influenza vaccination coverage in France during two influenza seasons (2007 and 2008) and during a context of pandemic influenza A(H1N1) in 2009 Vaccine 2011 29 4632 4637 10.1016/j.vaccine.2011.04.064 21550376

[B5] Lau JTF Yeung NCY Choi KC Cheng MYM Tsui HY Griffiths S Acceptability of A/H1N1 vaccination during pandemic phase of influenza A/H1N1 in Hong Kong: population based cross sectional survey BMJ 2009 339 b4164 10.1136/bmj.b4164 19861377 PMC2768779

[B6] Eastwood K Durrheim DN Jones A Butler M Acceptance of pandemic (H1N1) 2009 influenza vaccination by the Australian public The Medical Journal of Australia 2010 192 33 36 20047546 10.5694/j.1326-5377.2010.tb03399.x

[B7] Goodwin R Haque S Neto F Myers LB Initial psychological responses to Influenza A,H1N1 (“Swine flu”) BMC Infect Dis 2009 9 166 10.1186/1471-2334-9-166 19807908 PMC2765446

[B8] Zijtregtop EA Wilshut J Koelma N Van Delden JJ Stolk RP Van Steenbergen Broer J Which factors are important in adults’ uptake of a (pre)pandemic influenza vaccine ? Vaccine 2009 28 1 207 227 10.1016/j.vaccine.2009.09.099 19800997

[B9] Sestini P De Sario M Bugiani M Bisanti L Giannella G Kaisermann D Gruppo Collaborativo SIDRIA 2 Frequency of asthma and allergies in Italian children and adolescents: results from SIDRIA-2 Epidemiol Prev 2005 29 2 Suppl 24 31 16128550

[B10] Sacchetti R Gregori G Righi O Armani P Balduzzi P Boccellari R Il bambino e l’inquinamento atmosferico: la percezione del problema da parte dei genitori nella provincia di Piacenza Medico e Bambino 2004 6 399 400

[B11] Kass NE Otto J O’Brien D Minson M Ethics and severe pandemic influenza: maintaining essential functions through a fair and considered response Biosecu Bioterror 2008 6 3 227 236 10.1089/bsp.2008.0020 18795832

